# Hemophagocytic Lymphohistiocytosis Induced by Epstein-Barr Virus Infection and Newly Diagnosed Hodgkin Lymphoma

**DOI:** 10.7759/cureus.17752

**Published:** 2021-09-06

**Authors:** Ian Landry, Tamara Kurbanova, Ismail Omran, Khalid Mahmood

**Affiliations:** 1 Medicine, Icahn School of Medicine at Mount Sinai, Queens Hospital Center, New York, USA; 2 Internal Medicine, Icahn School of Medicine at Mount Sinai, Queens Hospital Center, New York, USA

**Keywords:** hemophagocytic lymphohistiocytosis (hlh), epstein- barr virus, classic hodgkin lymphoma, primary lymphoma, immune response

## Abstract

Hemophagocytic lymphohistiocytosis (HLH) is a rare, but life-threatening disorder of pathologic immune system activation which results in a hyperinflammatory state. Previous studies have suggested that hematologic malignancies are often inciting factors for HLH and portend a poorer prognosis. However, the substantial overlap between features of hematologic malignancies and HLH makes recognition and prompt diagnosis of HLH a complex and difficult task. We present a case of a young male who presented with acute dyspnea on exertion, unintentional weight loss, and fatigue. He was found to have pancytopenia, fever, splenomegaly, and Epstein-Barr viremia and was subsequently diagnosed with nodular sclerosing Hodgkin lymphoma. Five of eight 2004-HLH criteria were met and the patient was started on intravenous dexamethasone (10 mg/m^2^ daily), acyclovir, and AAVD (brentuximab, doxorubicin, vinblastine, dacarbazine) chemotherapy protocol with improvement in his symptoms and laboratory findings.

## Introduction

Hemophagocytic lymphohistiocytosis (HLH) is a rare, but life-threatening disorder in which an aberrant, severe systemic inflammatory syndrome develops. Acquired HLH disease is more commonly seen in adults and is often precipitated by viral infections (e.g., Epstein-Barr virus, cytomegalovirus), autoimmune disease, medications, or some types of malignancies, chiefly, lymphoma [1]. 

Initial studies in HLH led to the development of HLH-94 criteria which have subsequently been replaced by HLH-2004. Acquired HLH often presents as a sepsis-like syndrome that rapidly progresses to multi-organ failure. A typical presentation includes a variation in both clinical and laboratory signs. A diagnosis of acquired HLH may be made if a patient has five or more of the following criteria: fever, splenomegaly, and cytopenias (bicytopenia or pancytopenia), hyperferritinemia, hypertriglyceridemia, elevated soluble CD-25 (interleukin-2 receptor), or a low or absent natural killer cell activity. Additionally, patients may have associated transaminitis, elevated lactate dehydrogenase, and elevated D-dimer, with decreased levels of fibrinogen, albumin, and sodium [2,3]

Classical Hodgkin lymphoma (cHL) is a malignant disease of the lymphatic system often presenting in lymph node chains above the diaphragm and/or in the mediastinal nodes, most commonly in young (aged 20-40 years) males with previous Epstein-Barr viral infection [3]. Patients with cHL may present with classic B-cell symptoms; chiefly: fever, lymphadenopathy, night sweats, weight loss, and fatigue. Over 8,800 new cases of cHL are estimated in 2021 with a substantial cure rate of over 75% [4]. 

## Case presentation

A 49-year-old male with a significant past medical history of essential hypertension treated with hydrochlorothiazide and previous hernia repair presented with dyspnea on exertion, malaise, and fatigue for eight days prior to his admission. These symptoms were associated with an occasional non-productive cough, worse in the morning, and an unintentional weight loss of 8-lbs within the past month. At his baseline, his exercise tolerance was several miles of walking without stopping, but had notably decreased to less than two blocks due to dyspnea. He denied any fever or chills, diaphoresis, headaches, hematochezia, chest pain, palpitations, nausea, vomiting, diarrhea or urinary symptoms. He had no recent illnesses, sick contacts, or travel history. He denied receiving any recent vaccinations. He reported a sister with breast cancer currently undergoing treatment but denied any other family history of cancer, bleeding, or clotting disorders. The patient’s social history was significant for a former smoking history of approximately twenty pack-years, having quit nearly 3 years prior to his presentation, and a few shots of vodka or glasses of wine on the weekends. He denied any recreational drug use. He immigrated from Guyana in 2016 and is employed as a security guard in an apartment building. He was sexually active with one female partner and endorsed regular condom use. 

**Figure 1:**
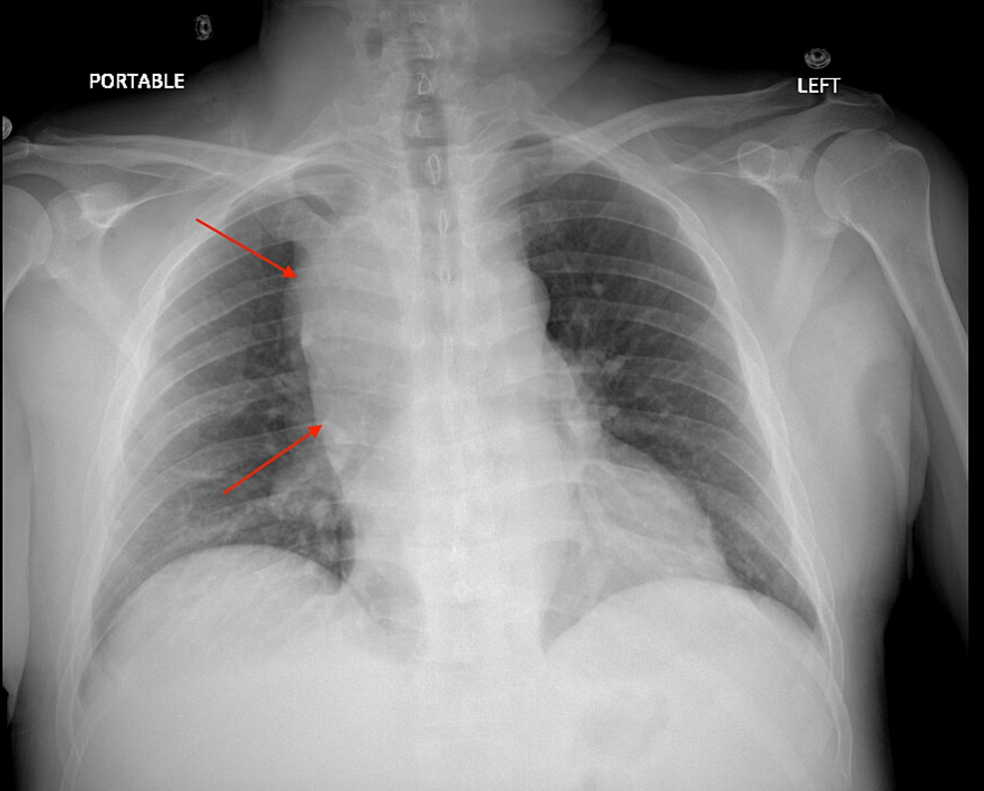
Chest X-Ray with large right-sided hilar mass

**Figure 2:**
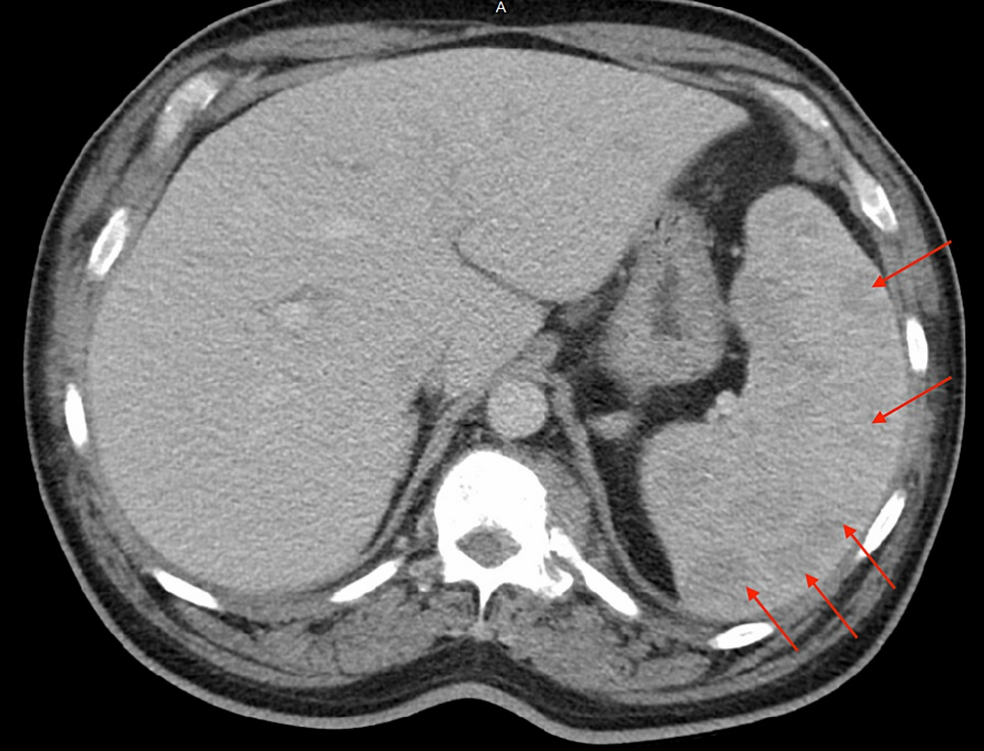
CT abdomen/pelvis showing splenomegaly with multiple hypodense lesions

**Figure 3:**
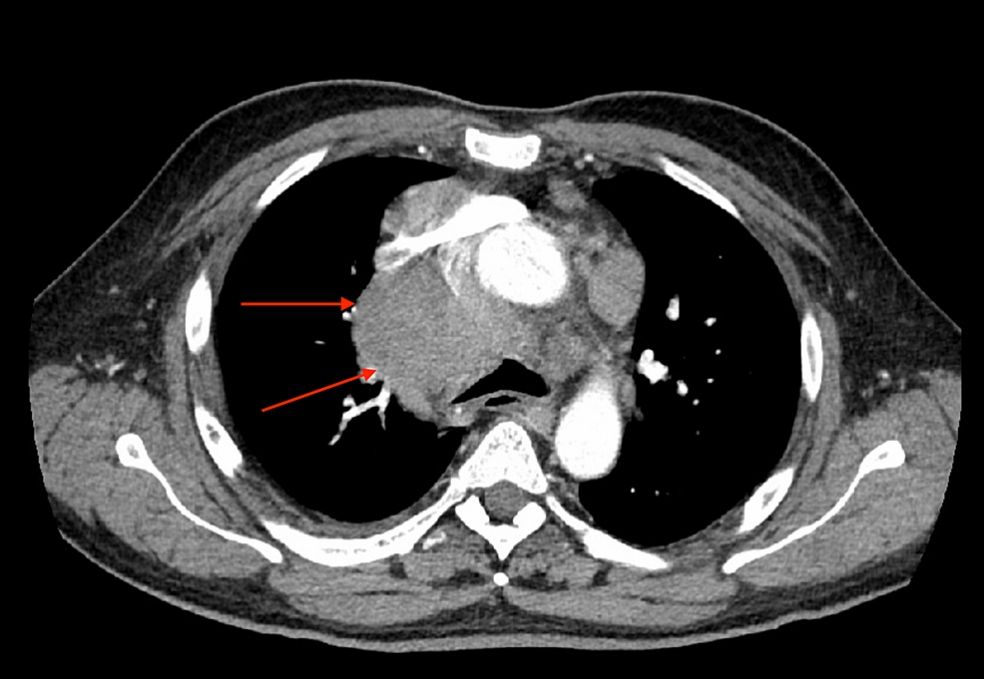
CT angiogram of the chest showing large mediastinal lymphadenopathy

Upon presentation to the emergency department, he was febrile to 100.6 °F and tachycardic to 126 bpm. Initial physical examination was significant for pallor, splenomegaly, and left-sided cervical lymphadenopathy. He was not in respiratory distress, had no petechiae or rash and was neurologically intact. Initial laboratory testing was significant for pancytopenia (Hgb 4.7 g/dL, WBC 2.10 x 103/mcL with 4% bandemia and absolute neutrophil count 1420-3250, platelets 111 x 103/mcL), hyponatremia 132 mmol/L, elevated pro-B-type natriuretic peptide (pro-BNP) 791 pg/mL, elevated D-dimer 850 ng/mL, mild hypoalbuminemia 3.1 g/dL, and transaminitis (aspartate aminotransferase [AST] 88 U/L, alanine aminotransferase [ALT] 103 U/L, alkaline phosphatase [ALP] 292 U/L). Troponin levels, renal function panel, and bilirubin levels were normal. Urinalysis was not significant. An initial chest X-Ray (Figure 1) showed a large, right-sided paratracheal/hilar opacity suspicious for mass versus adenopathy. CT abdomen/pelvis (Figure 2) was performed for transaminitis and splenomegaly which was significant for upper abdominal lymphadenopathy in the retroperitoneum and porta hepatis (largest 2.4 cm), bulky paraspinal masses in the upper abdomen, splenomegaly with multiple hypodense lesions, and sclerotic bony lesions. These findings were worrisome for metastatic disease. CT angiogram of the chest (Figure 3) was significant for marked splenomegaly with heterogenous enhancement due to multiple hypodense masses, no evidence of pulmonary emboli, and bulky abnormal mediastinal, hilar, lower neck, and upper abdominal lymphadenopathy. Echocardiogram showed a preserved ejection fraction of 55-60%. 

The patient received three units of packed red cells for symptomatic anemia with improvement in his hemoglobin to 7.8 g/dL. An anemia work-up was sent and was significant for severely elevated ferritin (4630 ng/mL), 17% iron, elevated haptoglobin (335 mg/dL), and elevated lactate dehydrogenase 342 U/L. Coagulation panel indicated prolonged prothrombin time (PT) (19.5 seconds) and activated partial thromboplastin time (aPTT) (41.6 seconds). Mixing studies were significant for abnormal aPTT study. The patient met sepsis physiology criteria with fever, leukopenia, and tachycardia. HIV, COVID-19 cepheid testing, and acute viral hepatitis panels were negative. Procalcitonin was slightly elevated at 0.27 ng/mL. A repeat complete blood count showed 9% bandemia. The patient was started on empiric antibiotic coverage with vancomycin and cefepime. Initial blood cultures returned with one of two bottles growing *Rothia aeria*, a gram-positive filamentous bacteria which often colonizes healthy individuals and was determined to be a contaminant by infectious disease consultation. Quantiferon-TB Gold returned indeterminate twice. Epstein-Barr virus (EBV) antibodies were sent which returned positive. Reflex polymerase chain reaction (PCR) was sent for EBV DNA and returned significantly elevated at 17,400 IU/mL. The patient continued to spike fever with T-max 103 °F despite negative cultures. Antibiotics were discontinued. Malignancy work-up was initiated with cancer antigen 125 (CA-125) elevated at 71 U/mL but with normal carcinoembryonic antigen (CEA) and carbohydrate antigen 19-9 (CA 19-9). The patient exhibited signs concerning for HLH (Table 1) and workup showed normal triglyceride levels (130 mg/dL) and severely elevated cluster of differentiation 25 (CD-25) soluble receptor (43,171 pg/mL). The patient underwent endoscopy with fine-needle aspiration (FNA) of a lower esophageal and porta hepatis lymph node. Interventional radiology performed a diagnostic biopsy of the mediastinal mass, which resulted in a diagnostically inadequate sample. Bone marrow biopsy with aspiration was attempted but resulted in a dry tap. Pathology from endoscopy-guided FNA returned significant for nodular sclerosing Hodgkin lymphoma. He was recommended for thoracic surgical evaluation and transferred to a tertiary care center for continued management. Repeat testing at the outside hospital (OSH) showed ferritin elevation as high as 11,000 ng/mL and the patient was started on intravenous dexamethasone (10 mg/m^2^ daily) for eight days with improvement in the ferritin levels to 4,000 ng/mL. Acyclovir 400 mg twice daily was started for the treatment of EBV viremia. Additionally, he was started on AAVD (brentuximab, adriamycin, vinblastine, and dacarbazine) chemotherapy protocol for the treatment of Hodgkin lymphoma, received port placement for pain control, and was discharged home with outpatient follow-up. At last check, the patient is tolerating therapy and the plan is to reevaluate his lymphadenopathy and viremia after several cycles of treatment. 

**Table 1:** HLH-2004 Criteria Source: Henter et al [2] HLH: hemophagocytic lymphohistiocytosis; CD-25: cluster of differentiation 25; NK-cell: natural killer cell

The Diagnosis of HLH may be established if either 1 or 2 below is fulfilled:
A molecular diagnosis consistent with HLH
HLH Diagnostic Criteria are met (requires 5 of 8 items)
HLH Criteria:
Fever
Splenomegaly
Cytopenias (at least 2 lineages must be affected)
Hypertriglyceridemia and/or hypofibrinogenemia
Hemophagocytosis in bone marrow, spleen. or lymph nodes
Low or absent NK-cell activity
Ferritin level >= 500 ug/L
Soluble CD-25 >= 2400 U/mL

## Discussion

HLH is a rare, albeit deadly, hyperimmune response that can often mimic septic shock due to its frequent association with multiple organ failure. Because of its rarity, HLH is often misdiagnosed, especially when it presents as a consequence of malignant hematology with overlapping symptoms. Our patient fulfilled five of eight 2004-HLH criteria and was found to have substantial EBV viremia and nodular sclerosing Hodgkin lymphoma, both of which are known triggers of HLH [5,6]. The prompt and correct diagnosis of HLH is important, as the delay in corticosteroids can lead to fatal end-organ damage and, if induced by malignancy, can delay or prevent chemotherapy induction. The goal of the treatment of HLH is to quickly suppress the massive immune response. Patients often undergo a series of weekly treatments with dexamethasone and etoposide with frequent monitoring of pro-inflammatory markers to assess for disease improvement. In patients with severe disease affecting the central nervous system, intrathecal preparations of methotrexate and hydrocortisone are used. In the select group of patients in whom these treatments are ineffective, experience relapse in disease, or have known mutations in the HLH gene, allogeneic hematopoietic cell transplantation is required [2]. 

While most of the literature in malignancy-associated HLH has been attributed to non-Hodgkin lymphoma [7], there is increasing evidence of classical Hodgkin lymphoma-associated HLH [8,9]. These findings suggest that an independent association between EBV and Hodgkin lymphoma is simultaneously influencing the development of an overactive immune state. A prevailing hypothesis suggests that an acute or reactivated infection in the setting of an immune system which is already downregulated by malignancy may result in an inappropriate response from the host immune system, with subsequent multi-organ destruction and often death. In fact, retrospective studies of patients with suspected HLH and malignancy have shown that those who meet the criteria for diagnosis often had worse immunosuppression (45% vs 33%, p=0.03) and a 20% early mortality rate (defined as death within one month) [10]. Similarly, an analysis of HLH patients subdivided by either lymphoma, systemic lupus erythematosus, or infectious associated, showed that 81% of the lymphoma-associated HLH patients died of multiple organ failure and disseminated intravascular coagulation, compared to only 12% of autoimmune or infection induced [11]. 

## Conclusions

All adult patients with cytopenia and fever who are found to have malignant conditions should be evaluated for HLH. Our patient initially presented with four obvious clinical or laboratory findings suggestive of HLH (fever, splenomegaly, cytopenia, and hyperferritinemia). In the setting of a newly discovered mediastinal mass and classic B-cell symptoms, a diagnosis of Hodgkin lymphoma was highly suspected. However, because HLH can mimic severe sepsis and/or septic shock, his initial treatment included broad-spectrum antibiotics for sepsis, a diagnosis that would preclude steroid treatment. Prompt hematologic consultation and consideration allowed for appropriate HLH work-up and diagnosis with cessation of antibiotics and treatment induction. Given the rarity of HLH, this hyperimmune state is not often considered when symptoms may be explained by more common entities. However, in patients with suspected lymphoma, it is necessary to properly diagnose HLH and elucidate any inciting factors, such as viral infections, which may cloud the diagnosis or worsen the prognosis. 
